# Chronic stress induces CD99, suppresses autophagy, and affects spontaneous adipogenesis in human bone marrow stromal cells

**DOI:** 10.1186/s13287-017-0532-3

**Published:** 2017-04-18

**Authors:** Zvenyslava Husak, Michael N. Dworzak

**Affiliations:** 1 St. Anna Kinderkrebsforschung, Children’s Cancer Research Institute, Zimmermannplatz 10, 1090 Vienna, Austria; 2 St. Anna Kinderspital, Kinderspitalgasse 6, 1090 Vienna, Austria

**Keywords:** Bone marrow stromal cells, CD99, Stress, MSC, Autophagy

## Abstract

**Background:**

Bone marrow-derived mesenchymal stromal cells (MSCs) are multipotent cells with a high constitutive level of autophagy and low expression of CD99. Under certain conditions, MSCs may develop tumorigenic properties. However, these transformation-induced conditions are largely unknown. Recently, we have identified an association between Hsp70, a main participant in cellular stress response and tumorigenesis, and CD99. Preliminary observations had revealed upregulation of both proteins in stressed long-term cultured MSCs. And so we hypothesized that CD99 is implicated in stress-induced mechanisms of cellular transformation in MSCs. Hence, we investigated the effects of prolonged stress on MSCs and the role of CD99 and autophagy in their survival.

**Methods:**

Human telomerase reverse transcriptase (hTERT) overexpressing immortalized MSCs and primary bone marrow stromal cells were used to investigate the influence of long-term serum deprivation and hypoxia on growth and differentiation of MSCs. Cell proliferation and apoptosis were evaluated using flow cytometry, differentiation capabilities of MSCs were assessed by immunohistochemical staining followed by microscopic examination. CD99, Hsp70 expression were analyzed using flow cytometry, western blotting, and reverse transcriptase polymerase chain reaction. Autophagy was explored with specific inhibitors using cell morphology examination and western blotting.

**Results:**

Chronic stress factors are able to change the morphology of MSCs and to inhibit spontaneous differentiation into adipocyte lineage. Furthermore, CD99 elevation and downregulation of p53 and p21 accompanied defective autophagy, which is usually associated with tumor formation. We found that inhibition of autophagy by chloroquine promoted cell detachment and modulated CD99 expression level whereas incorporation of CD99 recombinant protein into the cells suppressed autophagy.

**Conclusions:**

Obtained results provide a model for chronic stress-induced transformation of MSCs via CD99 and may therefore be highly relevant to mesenchymal tumorigenesis.

**Electronic supplementary material:**

The online version of this article (doi:10.1186/s13287-017-0532-3) contains supplementary material, which is available to authorized users.

## Background

Bone marrow-derived mesenchymal stromal cells (MSCs) are spindle-shaped nucleated cells with multilineage differentiation potential and immune-modulating ability [1]. They may develop into adipocytes, chondrocytes, and osteoblasts [1, 2]. Long-term survival of MSCs is one of their most significant characteristics. As a main strategy to maintain self-renewal and differentiation properties, MSCs use autophagy. This conserved catabolic mechanism to avoid cellular damage is a constitutive process in human MSCs where it also supports “stemness” properties [3]. On the other hand, MSCs are characterized by elevated transformation propensity [1]. Long-term expansion is under debate to lead to spontaneous transformation of MSCs [1, 4]. Among the main features of MSC transformation are changes in morphology, such as increased nucleus-to-cytoplasm ratio, disruption of cell cycle mechanism by p53 pathway(s) inhibition, and autophagy perturbation [5, 6]. Understanding the circumstances leading to the transformation of MSCs could be essential for therapeutic approaches.

MSCs have no specific surface markers and are usually recognized by co-expression of CD73, CD90, CD105, CD106, and CD146, and absence of hematopoietic markers such as CD34 and CD45 [1]. Previously, MSCs have been shown to be negative or only slightly positive in CD99, which otherwise is a widely expressed multifunctional surface molecule [7, 8]. Recently, however, we have identified an association between CD99 and Hsp70 – a main participant of cellular stress response [8]. This observation allowed us to hypothesize that CD99 is implicated in stress-induced signaling. MSCs are in the center of many studies investigating tumorigenesis under stress conditions, such as hypoxia and serum starvation. They are of special interest also because they are suggested to be the origin of Ewing’s tumors cells [7, 9], a highly aggressive pediatric malignancy characterized by high levels of CD99, presence of EWS-Fli1 rearrangement in most cases, and low levels of p53 and p21 [10, 11].

To investigate stress factors that influence stromal cells we mainly used a human bone marrow mesenchymal cell line immortalized with telomerase reverse transcriptase (TERT) [12]. These cells, like primary normal mesenchymal cells, maintain their growth rate, general functions, have a normal karyotype and phenotype, and are pluripotent. Advantages of using hTERT-positive MSCs are their long life span without senescence and the absence of donor-dependent heterogeneity. Previously we had used these cells to maintain survival of primary B cell precursor acute lymphoblastic leukemia (BCP-ALL) cells [13]. In the present study we found that prolonged stress conditions induce morphological transformation of MSCs, suppress autophagy, inhibit p53-p21 pathway, and upregulate CD99 – features which resemble well-described changes associated with neoplastic transformation of MSCs. Primary human bone marrow stromal cells were used in some experiments to verify the results obtained with the MSC cell line.

## Methods

### Cell culture

Human bone marrow stroma cell line immortalized by telomerase reverse transcriptase (TERT) overexpression (hMSCs) [12] was kindly provided by Dr. Giuseppe Gaipa (Centro Ricerca M. Tettamanti, Monza, Italy) and Dr. Dario Campana (St. Jude Children’s Research Hospital, Memphis, TN, USA). Primary bone marrow stroma cells were obtained from patients in remission after chemotherapy. Patients were recruited to the study (reference number 1500/2014), which was approved by the review board Ethikkommission der Universität Wien (University of Vienna Ethics Commission), and informed consent was obtained from parents or legal guardians for sampling procedures and subsequent laboratory investigations. Cells were maintained in RPMI medium (Fisher Scientific Austria, Vienna, Austria), supplemented with 10% fetal calf serum (FCS) (PAA Laboratories, Pasching, Austria), 2 mM glutamine and 100 U/ml antibiotic-antimycotic mix (both from LifeTech Austria, Vienna, Austria) at 37 °C and 5% CO_2_. To induce starvation, cells were washed with phosphate-buffered saline (PBS) and incubated in RPMI medium without FCS. For hypoxia experiments, cells were placed into an incubator with 1% oxygen for the required period of time. For the long-term assays, cells were plated in six-well plates and allowed to grow for 21 to 42 days, with the media changed every 3 days.

Cells were monitored in regular intervals by PCR to exclude mycoplasma infection.

### Antibodies, materials and reagents

The following anti-CD99 monoclonal antibodies (mAbs) were used for CD99 detection: DN16 (Scipac, Sittingbourne, UK) for western blotting (WB), phycoerythrin (PE)-conjugated 3B2 mAb (Caltag, Hamburg, Germany) for FACS, and CD99-FITC, and CD99-PE were from Becton Dickinson (BD) (San Jose, CA, USA). CD99 recombinant protein was from Abcam (Cambridge, UK). Hsp70 (W27) un-conjugated and PE-conjugated antibodies were from Santa Cruz Biotechnology (Dallas, TX, USA). Anti-glyceraldehyde-3-phosphate dehydrogenase (GAPDH), anti-BECN1, anti-p53, and anti-p21mAbs were from Santa Cruz, anti-microtubule-associated protein 1 light chain 3 (LC3), and anti-ATG13 from Sigma-Aldrich (Steinheim, Germany), and anti-PARP from Cell Signaling Technology (Leiden, The Netherlands). Goat anti-mouse IgG DYLight 800 and goat anti-rabbit IgG DY Light 800 antibodies were from THP Medical Products (Vienna, Austria). PE-conjugated goat anti-mouse IgG was purchased from Szabo-Scandic (Vienna, Austria). FITC- and APC-conjugated Annexin V, and amino-actinomycin D (7AAD) were from BD. Chloroquine (CQ), bafilomycin A1, pifithrin-alpha, Ponceau S-10, dexamethasone, isobutylmethylxanthine, insulin, indomethacin, ascorbic acid, β-glycerol phosphate, L-thyroxine, L-proline, insulin-transferrin-sodium selenite (ITS), sodium pyruvate, Oil Red O (ORO), Alizarin Red S (ARS), and Toluidine Blue (TB) all were from Sigma-Aldrich. PageRuler Prestained Protein Ladder was from Thermo Fisher Scientific Biosciences (St. Leon-Rot, Germany).

### Microscope imaging

Images were taken and processed using the Axiovert 40C inverted microscope (Zeiss, Göttingen, Germany) equipped with a MegaPixel FireWire PixeLink camera (PixeLink, Ottawa, ON, Canada).

### Flow cytometry

Stained cells were acquired with a FACS Calibur flow cytometer (BD). Data were analyzed using the CellQuest™ software (BD). Expression of cell antigens was quantified on the basis of mean fluorescence intensity (MFI) values as previously reported by us, using the CellQuest™ software [13]. Cytoplasmatic staining for CD99 and Hsp70 was performed with the Fixation/Permeabilization kit purchased from Invitrogen (Lofer, Austria) according to the manufacturer’s recommendations.

### Apoptosis, cell growth, cell cycle

Cells were scraped and resuspended in Annexin V binding buffer to incubate with Annexin V and 7AAD at room temperature (RT) for 20 minutes. Detection and quantification of apoptotic/necrotic cells was done by the flow cytometric analysis of labeled cells. The assay was performed according to the manufacturer’s instructions. Briefly, cells were collected from culture and then suspended in 100 μl Annexin V binding buffer (10 mM HEPES, pH7.4, 2.5 mM CaCl_2_, 140 mM NaCl) containing 1 μg/ml Annexin V. After 15 minutes incubation in the dark at RT, 7AAD (1 μl/tube) was added and apoptotic and dead cells were quantified on the flow cytometer.

Cell cycle analysis was assessed with the Cycletest Plus DNA Reagent kit (BD) according to the manufacturer’s protocol.

### Autophagy inhibition

For autophagy assay, cells were plated in six-well plates for 3 days in RPMI medium with or without FCS and 48 hours later were treated with or without CQ (75 μM and 150 μM) for 24 hours. Next, morphological examination and WB analysis were performed.

### Western blotting

Cells were washed with PBS, harvested, and centrifuged at 2000 rpm for 10 minutes. Laemmli sample buffer (4% SDS, 20% glycerol, 120 mM Tris-HCl, pH 6.8, 3% beta-mercaptoethanol, bromophenol blue) was used to prepare whole cell lysates. Total proteins were subsequently separated by 8.5 or 10% SDS-PAGE and transferred onto a nitrocellulose membrane (VWR, Radnor, PA, USA). After the transfer the membrane was evaluated in Ponceau S-10, washed with H_2_O and blocked in Western Blocking Reagent (Roche Diagnostics, Burgess Hill, UK) for 1 hour at RT. Immunoblot analysis was performed with the indicated antibodies and visualized by Odysseys Infrared Imager (Li-COR Biosciences, Lincoln, NE, USA).

### RT-PCR for CD99 and hsp70

Total RNA was isolated using RNeasy Micro Kit (Qiagen, Vienna, Austria). Single-strand cDNA was synthesized and hsc70/hsp70 and CD99 type I and type II were PCR amplified using the following specific primers: hsc70, forward: 5’-TGTGGCTTCCTTCGTTATTGG-3’ and reverse: 5’-GCCAGCATCATTCACCACCAT-3’; hsp70, forward: 5’-AGAGCCGAGCCGACAGAG-3’ and reverse: 5’-CACCTTGCCGTGTTGGAA-3’; GAPDH, forward: 5’-CCACTCCTCCACCTTTGAC-3’ and reverse: 5’-ACCCTGTTGCTGTAGCCA-3’ [8] (all from Eurofins MWG Operon, Ebersberg, Germany). CD99 type I and CD99 type II primers (all from VBS-Genomics, Vienna, Austria.), forward: 5’-GTGCGGCTAGCACCATGGCCCGCGGGGCTG-3’; CD99 type I reverse: 5’-CTAGTCTCGAGCTGGTAAGCAATGAAGCTAG-3’; CD99 type II reverse: 5’-GCTCTAGACCCTAGGTCTTCAGCCAT-3’ [14]. PCR products were separated on 2% agarose gel and visualized with GelStar Stain (Lonza, Rockland, ME, USA).

### Differentiation assays

Cells were incubated in 2 ml of the appropriate StemPro differentiation medium from Gibco Carlsbad, CA, USA (StemPro Adipogenesis Kit; StemPro Chondrogenesis Kit; StemPro Osteogenesis Kit) in six-well plates for 14 days. After 10 days, 500 μl of fresh medium were administrated to re-feed cell cultures.

### Adipocyte induction and detection

Cells were grown in six-well plates (VWR) in RPMI medium till they reached subconfluence. For long-term culture, we prepared an adipocyte-inducing medium based on Dulbecco’s modified Eagle’s medium (DMEM)-LC (LifeTech Austria) containing 1 μM dexamethasone, 500 μM isobutylmethylxanthine, 1 μg/ml insulin, and 100 μM indomethacin with supplementation. Before detection, cells were washed with PBS w/out Ca^2+^, Mg^2+^, fixed with 10% neutral-buffered formalin in PBS for 30–60 minutes and washed with water. Cells were incubated with 60% isopropanol for 5 minutes at RT. Afterward, isopropanol was removed and cells were stained with ORO working solution for 15 minutes at RT. After cell washing, hematoxylin solution was added for 1 minute. The washing step was repeated and cells were analyzed under an Axiovert 40C microscope.

### Osteocyte induction and detection

Osteocyte-induced differentiation medium was prepared using DMEM-LC containing 1 μM (10 nM) dexamethasone, 50 μg/ml ascorbic acid, 10 mM β-glycerol phosphate, and L-thyroxine with supplementation. Osteoblast detection was performed according to the standard protocol with some modification. Briefly, fixed cells were stained with 1 ml/well ARS working solution for 45 minutes, under gentle shaking, at RT, in the dark. Then unincorporated dye was aspirated, cells were washed with water, PBS was added, and the cells were analyzed under an inverted microscope.

### Chondrocyte induction and detection

For long-term chondrocyte induction we used Iscove’s modified Dulbecco’s medium (IMDM; LifeTech Austria) with 100 nM dexamethasone, 50 μg/ml ascorbic acid, 40 μg/ml L-proline, ITS, and 1 mM sodium pyruvate with supplementation. Cells were permeabilized with 0,5% Triton X-100 for 5 minutes, washed with water, and stained with TB working solution for 1–2 minutes. After washing, cells were microscopically analyzed.

### Statistical analysis

All numerical data including error bars represent the mean ± SEM. Statistical analysis was performed using Dunnett’s test and two-tailed Student’s *t* test.

## Results

### Serum starvation, hypoxia, and their combination change MSC phenotype

**Fig. 1:**
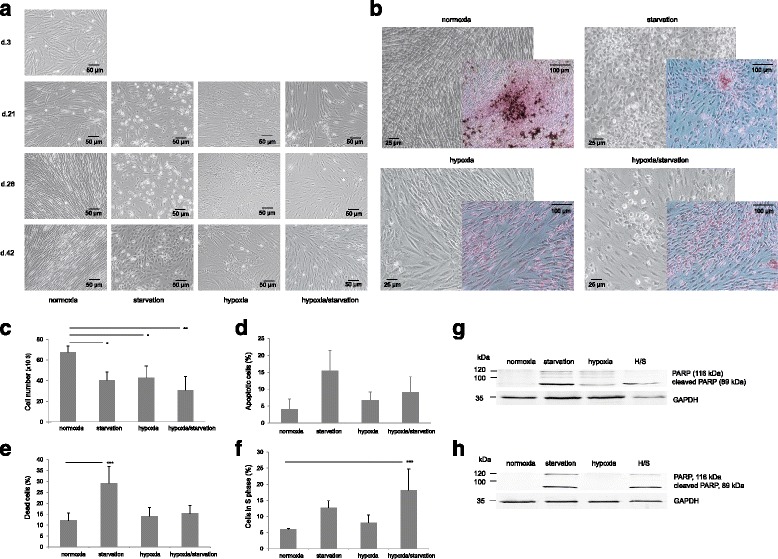
Stress changes morphology of MSCs and suppresses their spontaneous differentiation into adipocytes. **a** Microscopy pictures of time-dependent effects of serum starvation, hypoxia, and their combination on MSCs morphology. Cells were regularly observed under the microscope, photographs were taken at 3, 21, 28 and 42 days in culture. Pictures are representative data of six independent experiments. **b** Spontaneous differentiation of MSCs towards adipocytes, detected by Oil Red O at day 42 in normoxic culture, is much less prominent in starved, hypoxic, or combined hypoxic-starved (*H/S*) cultures. **c** Long-term (day 42) MSC culture growth measured by flow cytometry analysis (FACS). Only non-apoptotic (Annexin V-negative) cells are shown. From **c** to **f**: means with SEM of three experiments are shown. ^*^ *P* <0.1, ^**^ *P* <0.05, ^***^ *P* <0.01, Dunnett’s test. **d** Flow cytometry analysis of apoptosis in long-term MSC culture (Annexin V-positive cells are presented). **e** Cell death in long-term MSC culture (FACS). Staining was done by Annexin V and 7AAD, 7AAD-positive cells are shown. **f** Cell cycle analysis shows cells entered S phase at day 42 (FACS). Western blot analysis detects full-length PARP (116 kDa) and cleaved PARP fragment (89 kDa) in MSC cell line (**g**) and in primary stromal cells (**h**), thereby confirming apoptosis at day 42

First, we confirmed the potency of MSCs to develop into adipocytes, osteocytes, and chondrocytes by using respective cell culture differentiation mediums (from Gibco) (Additional file 1: Figure S1A). Next, we performed long-term culture experiments to investigate stress influence on used MSCs. Forty-two days exposure of MSCs to hypoxia (H) revealed a distinct morphological phenotype (Fig. 1a): flattened tri-to-polyangular cells with lower cell density and cobblestone areas as opposed to thread-“stretched” and compacted cells under oxygen supply (“normoxia”; i.e., cells cultured under normoxic conditions in medium supplemented with FCS). Serum starvation (S) induced shorter spindle-shaped and round cells with big nucleus. Combination of both stress factors, hypoxia and starvation (H/S), led to a mixed phenotype and thus illustrates the observation that hypoxia modulates starvation-induced effects on stroma cells [15]. To check the possibility of spontaneous differentiation of MSCs, specific stainings for adipogenic, osteogenic, and chondrogenic differentiation with Oil Red O, Alizarin Red S, and Toluidin Blue, respectively, were performed. We observed fat droplet accumulation in normoxia cultures detected by Oil Red O and could thus confirm spontaneous adipocyte differentiation of MSCs (Fig. 1b), which was not seen under stress conditions. After prolonged culture, cell numbers were the highest in normoxia and diminished under all stress conditions (Fig. 1c). To find the reasons for the difference, we examined apoptosis and proliferation of cells. Annexin V/7AAD staining showed increased cell death via apoptosis under starvation and mixed conditions (Fig. 1d and e). WB confirmed apoptotic death of long-stressed cells (Fig. 1g and h). Hypoxia did not differ from normoxia in these terms. Cell cycle analysis revealed more cells in S phase in starved and especially in mixed cultures (Fig. 1f). We concluded that stressed MSCs possess suppressed ability for spontaneous differentiation and demonstrate imbalance between apoptosis and proliferation. Experiments with primary stroma confirmed spontaneous adipocyte differentiation of long-term cultured cells and ability of stress to block it (Additional file 1: Figure S1B).

### Stress factors induce Hsp70

**Fig. 2:**
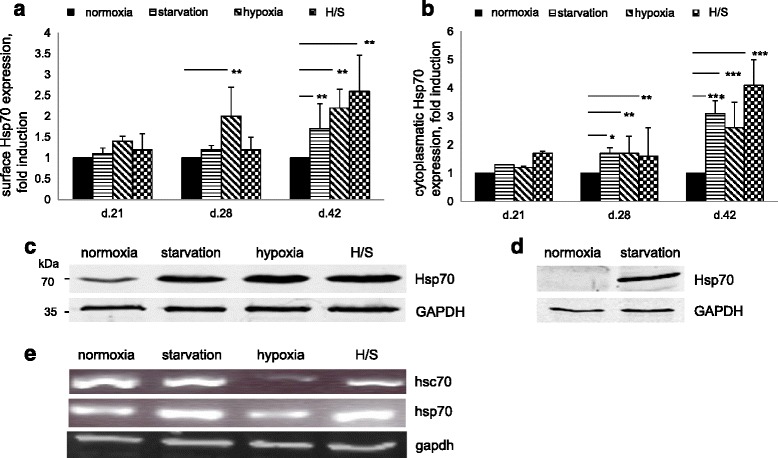
Hsp70 expression is modulated in MSCs under stress conditions. FACS data show time-dependent upregulation of (**a**) surface (s) Hsp70 and (**b**) cytoplasmatic (cy) Hsp70 by stress factors. Means with SEM of three experiments are shown. ^*^ *P* <0.1, ^**^ *P* <0.05, ^***^ *P* <0.01, Student’s *t* test. **c** Western blots of Hsp70 in normoxic and stressed MSCs at day 42. **d** WB shows Hsp70 upregulation in primary stromal cells (day 42). **e** RT-PCR of inducible (hsp70) and constitutive (hsc70) forms of Hsp70, gapdh (loading control)

On the surface of MSCs Hsp70 is present at very low levels (Fig. 2a). Under stress conditions, neither surface nor cytoplasmic levels of Hsp70 were elevated on MSCs (Fig. 2b) until 3 weeks in culture, but thereafter significant hypoxia-induced Hsp70 upregulation was detected at day 28 and day 42 (Fig. 2a and b). Starvation induced a later effect on MSCs, as only at day 42 was Hsp70 elevated (up to two times on the surface and more than three times in the cytoplasm). The combination of hypoxia and starvation proved synergistic in cytoplasmic Hsp70 upregulation (more than four times), but had no clear additional effect as compared to hypoxia alone on surface expression. Western blot analysis confirmed Hsp70 induction by stress factors both in MSC cell line (Fig. 2c) and in primary stromal cells (Fig. 2d). RT-PCR showed upregulation of induced hsp70 under starvation and combined conditions, but suppression of constitutive hsc70 in hypoxia and H/S cells (Fig. 2e). It suggests hypoxia-induced posttranscriptional regulation of Hsp70 expression.

### Chronic stress upregulates CD99

**Fig. 3:**
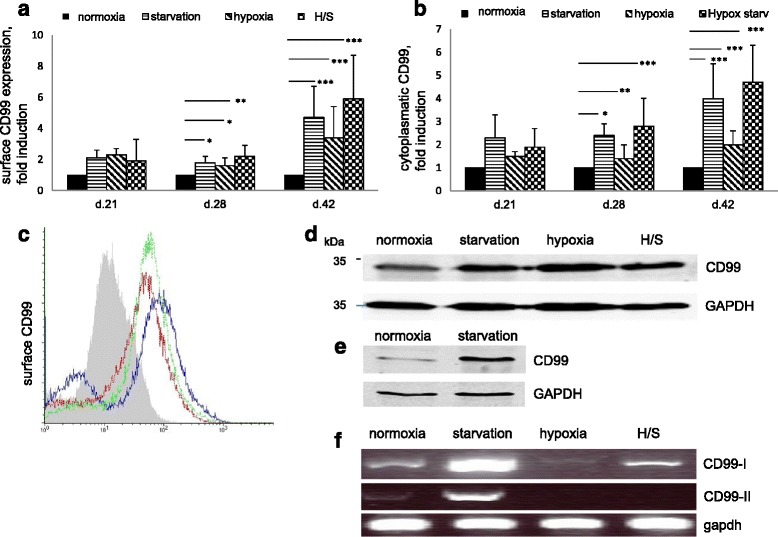
Time-dependent effects of stress on CD99 expression. **a** Flow cytometry analysis of surface (s) CD99 (recognized by 3B2 mAB) (mean of at least three experiments are shown with SEM) and (**b**) cytoplasmatic (cy) CD99. ^*^ *P* <0.1, ^**^ *P* <0.05, ^***^ *P* <0.01, Student’s *t* test. **c** Representative picture for sCD99 expression in normoxic (*gray profile*), starved (*blue line*), hypoxic (*red line*), and H/S cells (*green line*) cultures at day 42. Western blots of CD99 expression in MSC cell line (**d**) and in primary stromal cells (**e**). GAPDH – loading control. **f** RT-PCR analysis of both CD99 isoforms expression, type I and type II

Among the CD99 epitopes that we examined, only 3B2 was expressed at the detectable level. Three weeks under stress conditions revealed upregulation of CD99 both on the surface and in the cytoplasm (Fig. 3a-c). At day 42 surface CD99 was elevated more than four times under starvation, three times under hypoxia, and up to six times under combined treatment. A similar effect was observed in the cytoplasm. WB confirmed stress-induced upregulation of CD99 in MSCs on day 42 (Fig. 3d and e). RT-PCR analysis was performed to analyze expression of CD99 type I and type II isoforms [13] (Fig. 3f). Under normoxia, both forms were expressed with CD99 type I (long form) as the dominant one. Both CD99 forms were strongly induced by starvation but almost completely eliminated by hypoxia. Hence, CD99 protein overexpression is different in nature between stress conditions. While starvation increases CD99 production on the transcriptional level, hypoxia seemingly regulates CD99 on the posttranscriptional level, most probably by stabilizing the existing protein.

### Stress inhibits autophagy in MSCs

**Fig. 4:**
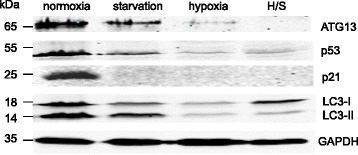
Chronic stress suppresses autophagy in MSCs. Western blot shows inhibition of LC3 (an autophagy marker) by prolonged (42 days) stress in MSC cell line. p53 and p21 are also delayed by stress. ATG13, another autophagy marker, is strongly downregulated in MSCs

Autophagy is constitutively active in MSCs [3], stabilizing survival and self-renewal. A common way to monitor autophagy is to compare ratio of LC3-I (a cytosolic non-active form of LC3 autophagy marker) and LC3-II (an active form in autophagosome membrane) as well as detect total level of LC3 under control and experimental conditions by using western blot analysis. We found that prolonged hypoxia alone and in combination with starvation strongly diminished autophagy, as revealed by downregulation of total LC3 (Fig. 4). Starvation, hypoxia, and their combination suppressed production of LC3-I and LC3-II. Additionally, ATG13, another autophagy marker, was also found to be reduced by stress factors. In parallel, p53 and p21, which are both also implicated in autophagy [16], were even more pronouncedly inhibited by prolonged stress factors. Prolonged stress (starvation) induced autophagy inhibition in primary stromal cells too (Additional file 2: Figure S2).

### Autophagy inhibitor promotes detachment of MSCs and modulates CD99

**Fig. 5:**
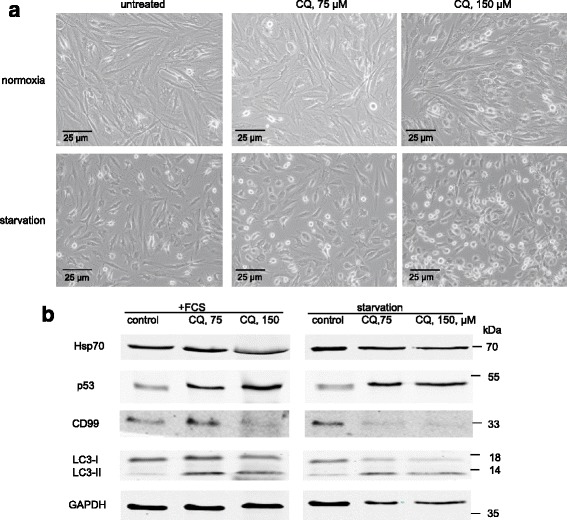
Chloroquine (CQ) promotes cells detachment and affects CD99. **a** Microscopy pictures of CQ-induced changes in cell culture: morphological transformation and detachment of cells. MSCs were cultured in starvation medium for 3 days. After the first 48 hours in culture, CQ (75 μM and 150 μM) was added for the next 24 hours. **b** Western blot of extracts from CQ-treated control (normoxic) and starved MSCs. Representative data of three independent experiments are shown

To shed light on the role of autophagy in the survival of MSCs, normoxic and starved MSCs were incubated for 3 days, with or without the inhibitor of autophagy, i.e., chloroquine (CQ), for the last 24 hours. Autophagy abrogation by CQ induced cell detachment and round cell morphology in non-stressed and starved cultures in a dose-dependent manner (Fig. 5a; Additional file 3: Figure S3). Simultaneously, WB analysis showed upregulation of p53 by CQ in normoxia and starved cells (Fig. 5b). Hsp70, on the other hand, was downregulated as well as CD99. However, lower doses of CQ (75 μM) induced CD99 in normoxic cells. Thus, autophagy regulates morphology and attachment ability of MSCs, and affects p53 as well as CD99 and Hsp70.

### CD99 modulation inhibits autophagy in MSCs

**Fig. 6:**
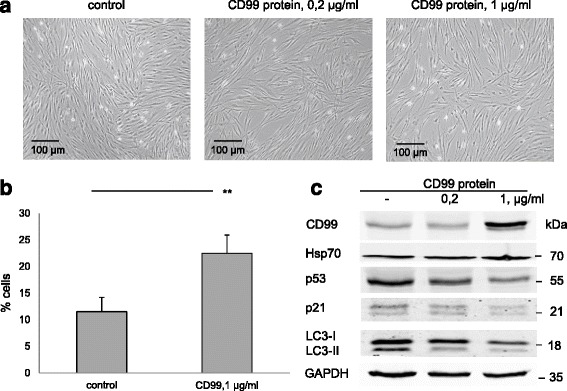
CD99 is a negative regulator of autophagy in MSCs. **a** CD99 recombinant protein had no influence on cell morphology after 3 days in culture but induced proliferation of MSCs. **b** Cell cycle analysis is shown. Means with SEM of three experiments are shown. ^**^ *P* <0.05, Student’s *t* test. **c** Autophagy machinery – p53, p21, and LC3 – was suppressed in a dose-dependent manner by introduction of CD99 recombinant protein (WB analysis)

Since CD99 upregulation accompanies chronic stress-induced autophagy inhibition, we asked whether CD99 might regulate autophagy. Administration of recombinant CD99 protein did not change cell morphology (Fig. 6a), but did promote S phase cell entry (Fig. 6b). Incorporation of recombinant CD99 into MSCs decreased p53 and p21 expression as well as LC3-I and LC3-II in the cells (Fig. 6c). These results suggest a CD99-dependent downregulation of autophagy in MSCs.

### Hypoxia and starvation suppress differentiation potential of hMSCs

**Fig. 7:**
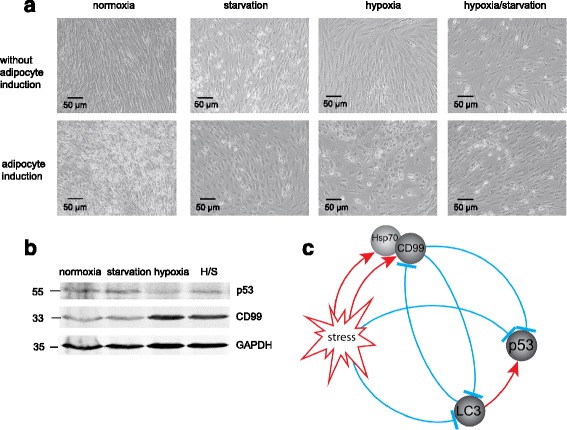
Stress factors inhibit induced differentiation of MSCs. **a** Adipocyte differentiation induced for 14 days modulates cell morphology more prominently as spontaneous differentiation does after 42 days. **b** Western blot of extracts from MSC cells induced to adipocytes shows upregulation of CD99 under stress conditions. **c** Schematic presentation of stress-induced pathways in MSCs. *Arrow-headed red lines* and *bar-headed blue lines* indicate activation and inhibition, respectively

MSCs were cultured under stress conditions (hypoxia, starvation, and their combination) for 11 days to achieve visible morphological changes of cells and then adipogenic, osteogenic, and chondrogenic differentiation was induced with the appropriate mediums. After a further 14 days, specific stainings with Oil Red O, Alizarin Red S, and Toluidin Blue, respectively, were performed. In the normoxic cultures, MSCs were induced to adipocytes and osteocytes, as well as less prominently to chondrocytes (Additional file 4: Figure S4). Adipocyte-induced MSCs in stressed cultures developed round morphology (Fig. 7a) similar to cells under corresponding conditions at day 42 (see Fig. 1a). Adipogenesis of stressed primary stromal cells was blocked too (Additional file 5: Figure S5). In MSCs induced to adipocytes, CD99 expression was elevated by hypoxia and mixed conditions, while p53 was downregulated (Fig. 7b). Obtained results reflected the pattern of MSCs spontaneously differentiated toward adipocyte lineage.

## Discussion

Interest in MSCs as a source of stem cells for regenerative medicine arises due to their ability to differentiate into multiple lineages. Many efforts are currently focusing on the investigation of the transformation potentials of MSCs, either primary or immortalized. Conflicting results show, on the one hand, the stable genotype and phenotype of MSCs in long-term (12 weeks) cultures [4], and on the other, spontaneous neoplastic transformation of MSCs [17]. Even cell lines immortalized in the same way – by overexpression of hTERT, for example – are reported to be either genomically instable [18] and transformed [19] or stable [12], with unclear transforming potential. Generally, hTERT overexpression and also loss of p53 and p21 are on the top of the list of events shown to be the most relevant for induction of MSC transformation [5, 20–22]. The circumstances that induce these mutations and lead to the critical changes in MSCs are, however, still unknown. According to some hypotheses they may be promoted by external factors.

Previously, different stress stimuli were tested for their effects on MSCs. It was shown that stressed MSCs lose their phenotype stability and change differentiation potentials: heat shock enhanced osteoblast differentiation of hTERT-immortalized human MSCs [23]; hypoxia increased ectodermal differentiation into neuron-like cells [15], but reduced adipogenic and osteogenic differentiation of primary human MSCs [15, 24]; serum starvation caused upregulation of myocardial markers on human bone marrow-derived MSC [25] and epithelial markers on adipose stem cells [26]; exposure to electric field or to mechanical strain improved hMSCs osteogenic differentiation [27] and pulsed electromagnetic fields induced chondrogenesis [28]; high cell density initiated commitment of hMSCs to endothelial cells [29]. To find conditions which may cause in vitro transformation of MSCs, we decided to examine the influence of typical stress factors, like starvation and hypoxia, on a hTERT-positive MSC cell line.

It is well known that oxygen distribution in different tissues is uneven, with 1% to 7% gradient across bone marrow, for example. However, survival of MSCs after transplantation is not supported by conditions associated with hypoxia and starvation, like ischemia. Taking into account that usual in vitro cell cultures are performed under 21% oxygen is an important issue to investigate characteristics of MSC cells over prolonged periods of time [15, 24].

First, we observed that long-term stressed MSCs exhibited drastic changes in morphology, especially in cultures which underwent starvation or combined H/S conditions: they showed increased nucleus-to-cytoplasm ratio in round cells or flat polyangular cells in contrast to stretched normoxic cells. The latter proliferated faster than others but at day 21 cells reached confluence, and by day 42 their proliferation was strongly suppressed as was seen from cell cycle analyses. Normoxic cells exhibited the lowest level of apoptosis, starved cells, the highest (around 30% more). As expected, apoptosis was not elevated by hypoxia and was less visible when hypoxia was combined with starvation [15].

As stress-induced morphological changes in MSCs were observed, we looked for further evidences of possible transformation: altered differentiation, changes in metabolism, or stress-dependent protein modulation.

We found that multipotent hMSCs underwent spontaneous adipogenic differentiation after a few weeks in normoxic but not in stressed cultures. This could, of course, also be caused by the aging of very dense cells, similar to the influence of chemotherapy or irradiation on bone marrow stromal cells [30, 31]. However, activity of autophagy and cell cycle machinery, which are shown to be age-related [32, 33], as well as Hsp70 and CD99 expression levels, were unchanged. Hsp70 is involved in cellular transformation (reviewed in ref. [34]) and recently has been recognized as a negative regulator of autophagy [35]. CD99 expression depends on the differentiation stage of many cell types [13, 36–38], serves by itself as inhibitor of differentiation [36, 37], and modulates survival of different cells [13, 39]. That is why the obtained results are probably indicative of amplification of cell density-dependent signals directing MSCs along adipocyte lineage, on the one hand, and “stemness-saving” signals of some stress stimuli [15, 24], on the other hand. Indeed, high cell density is required for MSCs differentiation into each of the three lineages (adipocytes, osteocytes, or chondrocytes) [40], but spontaneous differentiation just into adipocyte lineage results from a combined effect of cell density and choice of medium. Moreover, the requirement of autophagy for adipogenesis has been described recently, linking LC3 expression and lipid droplets formation (reviewed in refs. [41, 42]). However, autophagy is also involved in osteogenesis [43] and chondrogenesis [44] and, therefore, should be one of the key regulators of mesenchymal development. Here we found that autophagy, which is a constitutive process in MSCs [3], was dramatically reduced by stress.

It is widely known that tumorigenesis may be promoted either by suppression of autophagy or by autophagy induction (reviewed in ref. [45]). This duality can be explained by the indispensable role of autophagy for normal cell homeostasis where deregulation of balance leads to cell transformation. Here we found that inhibition of autophagy by chloroquine in a dose- and time-dependent manner induced morphological changes of the MSCs, followed by detachment and cell death, thus mimicking stress effects. However, CQ upregulated p53 as well as downregulated CD99 and Hsp70. The discrepancy between pro-apoptotic CQ effects and upregulation of CD99 and Hsp70 in chronically stressed cells suggests involvement of both proteins into a pro-survival adaptation strategy of MSCs. Indeed, we found that overexpression of CD99 promoted proliferation of MSCs. It has been shown that hypoxia induces upregulation of both CD99 [46] and Hsp70 [47]. These two proteins may be regulated by the hypoxia-inducible factor (HIF) system. Increased expression of HIF-alpha in hypoxic MSCs [48], Hsp70-HIF-1alpha interactions under hypoxic conditions [49], as well as CD99 targeting by von Hippel-Lindau tumor suppressor gene (VHL) through HIF-alpha subunits [46], have been described in the literature. Additionally, we found that treatment with bafilomycin A1, another autophagy inhibitor, revealed not only inhibition of autophagy detected by LC3 but induction of apoptosis machinery too (Additional file 6: Figure S6).

Furthermore, stress-induced changes of MSC phenotype and molecular pathways correlated with CD99 upregulation. At each examined time point, the CD99 level in normoxic cells was lower than in stressed cells. RT-PCR and WB showed that this effect was due to stabilization of CD99 under stress-like hypoxia and under starvation due to its upregulation. In parallel, induction of Hsp70 was observed. To find a direct link between CD99 and MSCs transformation, CD99 recombinant protein was administered to the cell cultures and the level of autophagy was examined. We observed CD99-dependent autophagy delay (suppression of p53, p21, and LC3) paralleled by cell cycle promotion. A similar effect, which is a hallmark of transformation (inhibition of p53 and p21), was seen under stress. Hence, our results demonstrate the interplay between CD99 and autophagy. Recently, we have already shown that CD99 is a functional partner for Hsp70 [8], and Hsp70 has been suggested to be a negative regulator of autophagy [35]. Direct delivery of Hsp70 into MSCs protected the cells from hypoxia-induced apoptosis [50]. We speculate that upregulation of CD99 and inhibition of adipocyte differentiation under stress might be linked to Sp1 transcriptional activity. Sp1 was shown to be a negative regulator of adipogenesis in MSCs [51], and a positive regulator of CD99 [52].

Altogether, the presented data demonstrate the ability of chronic stress to transform the morphology of MSCs, to suppress differentiation, to alter cell proliferation, to inhibit autophagy, and to induce apoptosis. We also showed that CD99 and Hsp70 elevation and disappearance of p53 and p21 accompany defected autophagy. While short-term inhibition of autophagy by chloroquine downregulates CD99 expression in MSCs and induces p53, on the one hand, long-term stress in MSCs leads to CD99 upregulation and p53 suppression, on the other. Thus, we hypothesize that CD99 may serve as a pro-survival molecule in stressed MSCs, which in cooperation with Hsp70 adapts them to unfavorable conditions. This suggestion is supported by the experiment in which modulation of CD99 level by CD99 recombinant protein inhibits LC3, an autophagy marker, and blocks the p53-p21 pathway (Fig. 7c).

## Conclusions

Our results provide a model of spontaneous adipocyte differentiation of long-term cultured hTERT-immortalized MSCs as well as primary stromal cells and show chronic stress to be a differentiation blocking mechanism potentially leading to transformation of MSCs along with autophagy inhibition in a CD99-dependent manner. These circumstances should be taken into account, among others [53], in the current efforts for standardization of MSC therapy.

## Additional files

Additional file 1: Figure S1. Induced differentiation of MSCs. (A) hTERT + MSCs may develop into adipocytes, osteocytes, and chondrocytes. Microscopy pictures of the effects of respective cell culture differentiation mediums on MSC cell line. Specific stainings with Oil Red O (for adipocytes), Alizarin Red S (osteocytes), and Toluidin Blue (chondrocytes) were performed. Cells were regularly observed under the microscope, photographs were taken at 14 days in culture. Pictures are representative data of six independent experiments. (B) Spontaneous adipocyte differentiation of primary stromal cells. Primary stromal cells were stained with Oil Red O to detect adipocyte differentiation. Photographs were taken at day 21 in culture. Pictures are representative of four independent experiments. (PPTX 6043 kb)

Additional file 2: Figure S2. Stress inhibits autophagy in primary stromal cells. Western blot shows inhibition of LC3 (autophagy marker), p53 and p21 by prolonged (42 days) stress in primary stromal cells. (PPTX 74 kb)

Additional file 3: Figure S3. Chloroquine (CQ) induces morphological changes of primary stromal cells and their detachment. Primary stromal cells were cultured in starvation medium for 3 days, with CQ for the last 6 hours. Representative pictures of three independent experiments are shown. (PPTX 2986 kb)

Additional file 4: Figure S4. Stress conditions affect induced differentiation of MSCs. MSCs were cultured under stress conditions (hypoxia, starvation, and their combination) for 11 days to achieve visible morphological changes of cells and then adipogenic, osteogenic, and chondrogenic differentiation was induced with the appropriate mediums. After a further 14 days, specific stainings with Oil Red O, Alizarin Red S, and Toluidin Blue, respectively, were performed. (PPTX 2438 kb)

Additional file 5: Figure S5. Starvation blocks induced adipogenesis of primary stromal cells. Prolonged stress (starvation) blocks induced adipocyte differentiation of primary stromal cells. ORO staining (*lower panel*) was performed at day 21. Representative pictures of four independent experiments. (PPTX 3141 kb)

Additional file 6: Figure S6. Bafilomycin A1 treatment of MSCs. (A) hTERT-positive MSCs were starved for 3 days and treated with bafilomycin A1 for the last 3 hours. Pictures are representative of three independent experiments. (B) MSCs treated with bafilomycin A1 were subjected to WB to detect autophagy-involved proteins. (PPTX 1761 kb)

## Abbreviations

7AAD: amino-actinomycin D
ARS: Alizarin Red S
BCP-ALL: B cell precursors acute lymphoblastic leukemia
CQ: Chloroquine
DMEM: Dulbecco’s modified Eagle’s medium
FCS: Fetal calf serum
GAPDH: Glyceraldehyde-3-phosphate dehydrogenase
HIF: Hypoxia-inducible factor
hMSC: Human bone marrow mesenchymal stromal cells
hTERT: Human telomerase reverse transcriptase
IMDM: Iscove’s modified Dulbecco’s medium
ITS: Insulin-transferrin-sodium selenite
LC3: Microtubule-associated protein 1 light chain 3
ORO: Oil Red O
PBS: Phosphate-buffered saline
TB: Toluidine Blue
WB: Western blotting
