# Supplementary material for: Chronic stress induces CD99, suppresses autophagy, and affects spontaneous adipogenesis in human bone marrow stromal cells
Source: Stem Cell Res Ther. 2017 Apr 18;8:83. doi: 10.1186/s13287-017-0532-3 (PMC5395812; doi:10.1186/s13287-017-0532-3)
Supplement: Supplementary file 1 — Induced differentiation of MSCs. (A) hTERT + MSCs may develop into adipocytes, osteocytes, and chondrocytes. Microscopy pictures of the effects of respective cell culture differentiation mediums on MSC cell line. Specific stainings with Oil Red O (for adipocytes), Alizarin Red S (osteocytes), and Toluidin Blue (chondrocytes) were performed. Cells were regularly observed under the microscope, photographs were taken at 14 days in culture. Pictures are representative data of six independent experiments. (B) Spontaneous adipocyte differentiation of primary stromal cells. Primary stromal cells were stained with Oil Red O to detect adipocyte differentiation. Photographs were taken at day 21 in culture. Pictures are representative of four independent experiments. (PPTX 6043 kb) [file 13287_2017_532_MOESM1_ESM.pptx]

## Slide 1
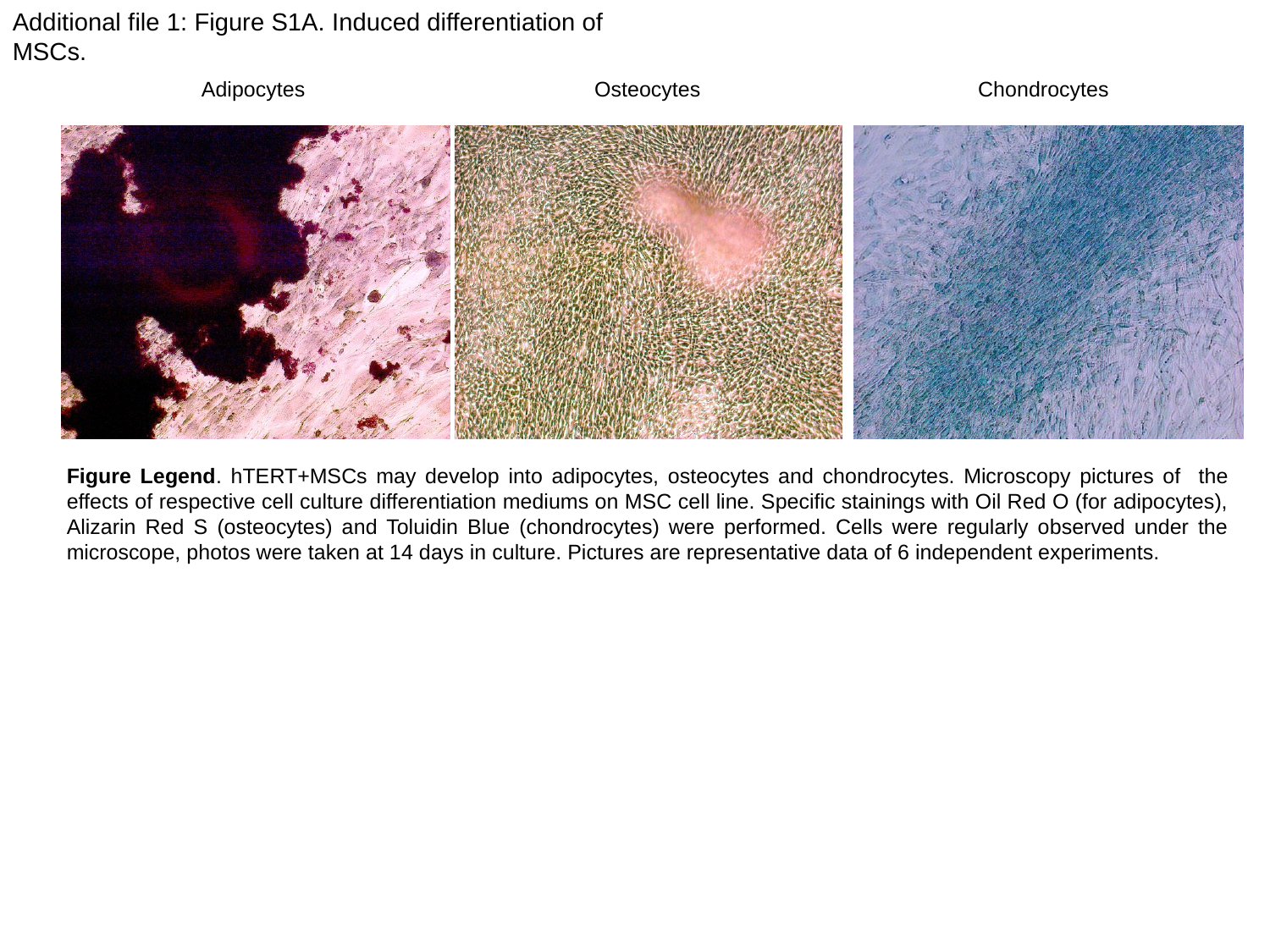

Additional file 1: Figure S1A. Induced differentiation of MSCs.
 Adipocytes Osteocytes Chondrocytes
Figure Legend. hTERT+MSCs may develop into adipocytes, osteocytes and chondrocytes. Microscopy pictures of the effects of respective cell culture differentiation mediums on MSC cell line. Specific stainings with Oil Red O (for adipocytes), Alizarin Red S (osteocytes) and Toluidin Blue (chondrocytes) were performed. Cells were regularly observed under the microscope, photos were taken at 14 days in culture. Pictures are representative data of 6 independent experiments.

## Slide 2
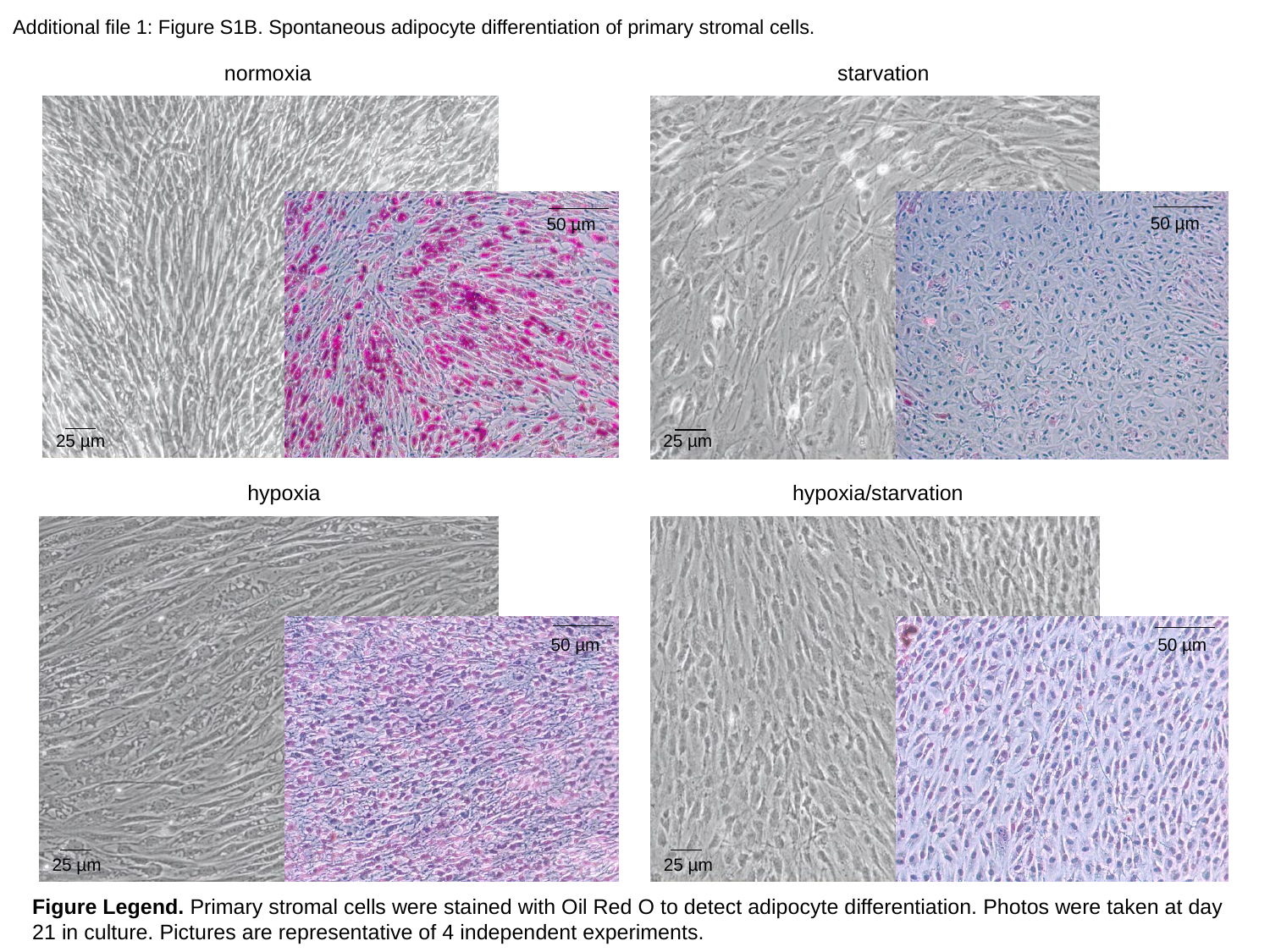

# Additional file 1: Figure S1B. Spontaneous adipocyte differentiation of primary stromal cells.
starvation
normoxia
50 µm
50 µm
25 µm
25 µm
hypoxia
hypoxia/starvation
50 µm
50 µm
25 µm
25 µm
Figure Legend. Primary stromal cells were stained with Oil Red O to detect adipocyte differentiation. Photos were taken at day 21 in culture. Pictures are representative of 4 independent experiments.
