# Supplementary material for: Chronic stress induces CD99, suppresses autophagy, and affects spontaneous adipogenesis in human bone marrow stromal cells
Source: Stem Cell Res Ther. 2017 Apr 18;8:83. doi: 10.1186/s13287-017-0532-3 (PMC5395812; doi:10.1186/s13287-017-0532-3)
Supplement: Supplementary file 2 — Stress inhibits autophagy in primary stromal cells. Western blot shows inhibition of LC3 (autophagy marker), p53 and p21 by prolonged (42 days) stress in primary stromal cells. (PPTX 74 kb) [file 13287_2017_532_MOESM2_ESM.pptx]

## Slide 1
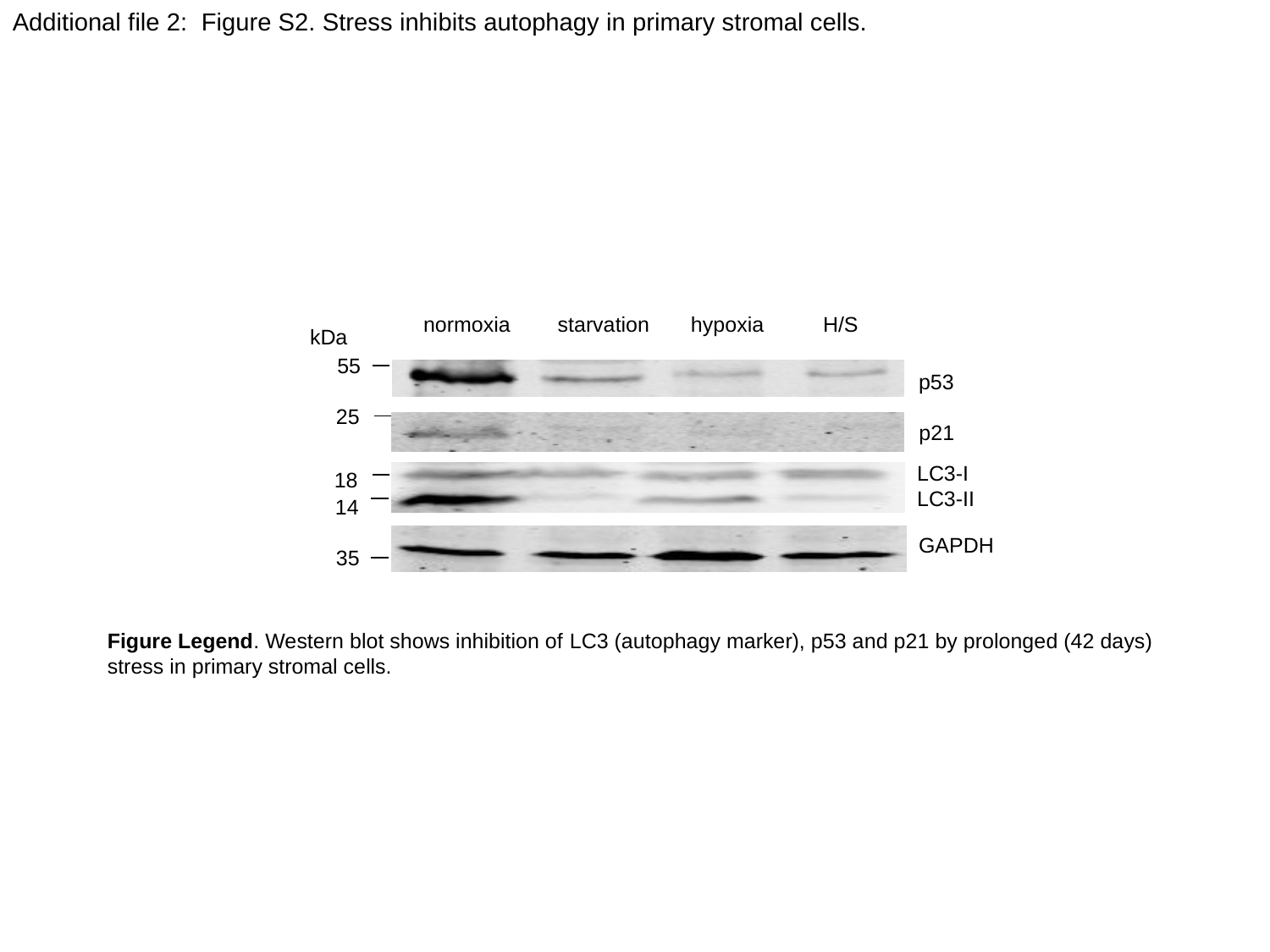

Additional file 2: Figure S2. Stress inhibits autophagy in primary stromal cells.
normoxia starvation hypoxia H/S
kDa
55
p53
25
p21
LC3-I
LC3-II
18
14
GAPDH
35
Figure Legend. Western blot shows inhibition of LC3 (autophagy marker), p53 and p21 by prolonged (42 days) stress in primary stromal cells.
