# Supplementary material for: Chronic stress induces CD99, suppresses autophagy, and affects spontaneous adipogenesis in human bone marrow stromal cells
Source: Stem Cell Res Ther. 2017 Apr 18;8:83. doi: 10.1186/s13287-017-0532-3 (PMC5395812; doi:10.1186/s13287-017-0532-3)
Supplement: Supplementary file 3 — Chloroquine (CQ) induces morphological changes of primary stromal cells and their detachment. Primary stromal cells were cultured in starvation medium for 3 days, with CQ for the last 6 hours. Representative pictures of three independent experiments are shown. (PPTX 2986 kb) [file 13287_2017_532_MOESM3_ESM.pptx]

## Slide 1
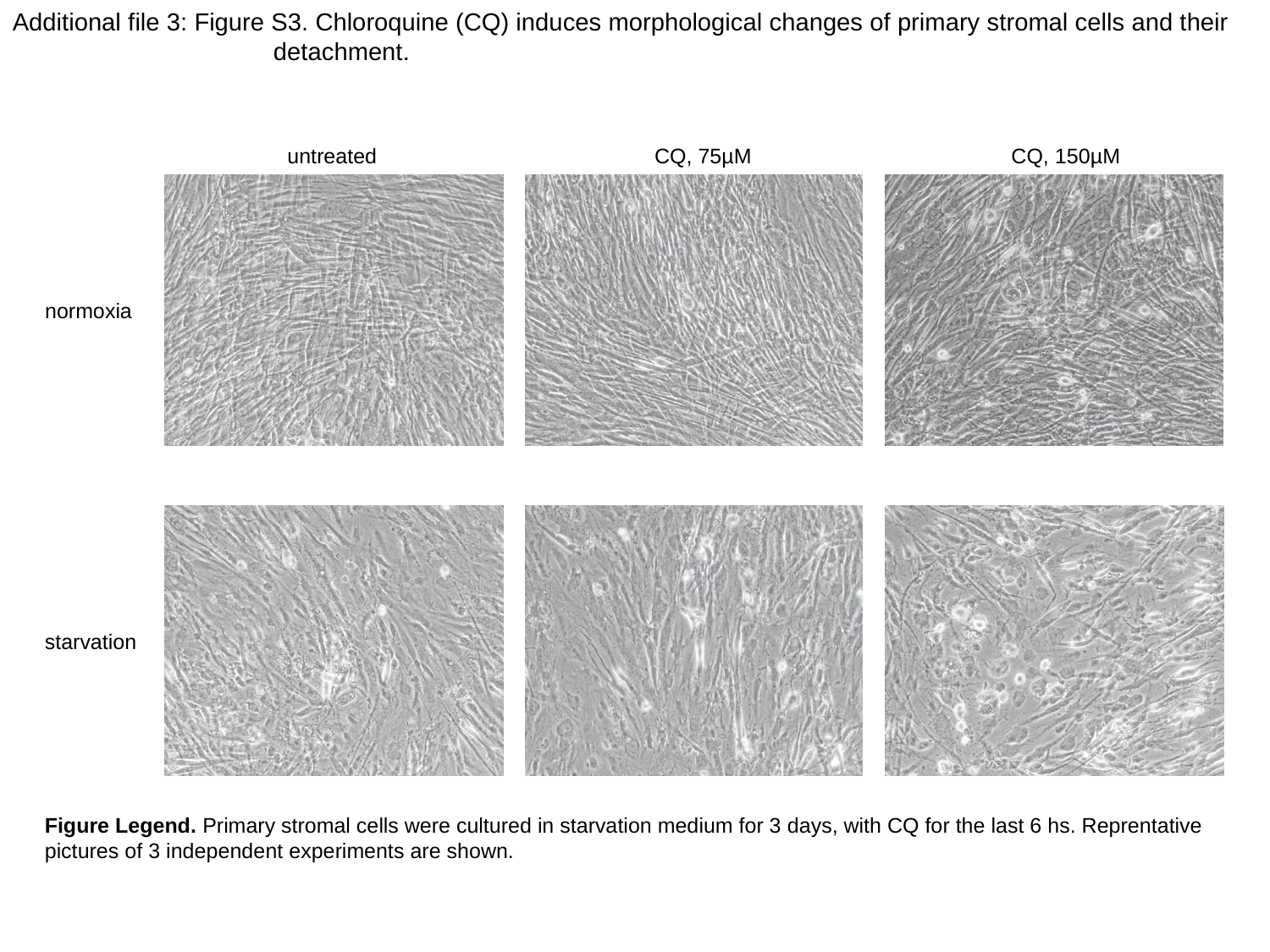

Additional file 3: Figure S3. Chloroquine (CQ) induces morphological changes of primary stromal cells and their 		 detachment.
untreated CQ, 75µM CQ, 150µM
normoxia
starvation
Figure Legend. Primary stromal cells were cultured in starvation medium for 3 days, with CQ for the last 6 hs. Reprentative pictures of 3 independent experiments are shown.
