# Supplementary material for: Chronic stress induces CD99, suppresses autophagy, and affects spontaneous adipogenesis in human bone marrow stromal cells
Source: Stem Cell Res Ther. 2017 Apr 18;8:83. doi: 10.1186/s13287-017-0532-3 (PMC5395812; doi:10.1186/s13287-017-0532-3)
Supplement: Supplementary file 4 — Stress conditions affect induced differentiation of MSCs. MSCs were cultured under stress conditions (hypoxia, starvation, and their combination) for 11 days to achieve visible morphological changes of cells and then adipogenic, osteogenic, and chondrogenic differentiation was induced with the appropriate mediums. After a further 14 days, specific stainings with Oil Red O, Alizarin Red S, and Toluidin Blue, respectively, were performed. (PPTX 2438 kb) [file 13287_2017_532_MOESM4_ESM.pptx]

## Slide 1
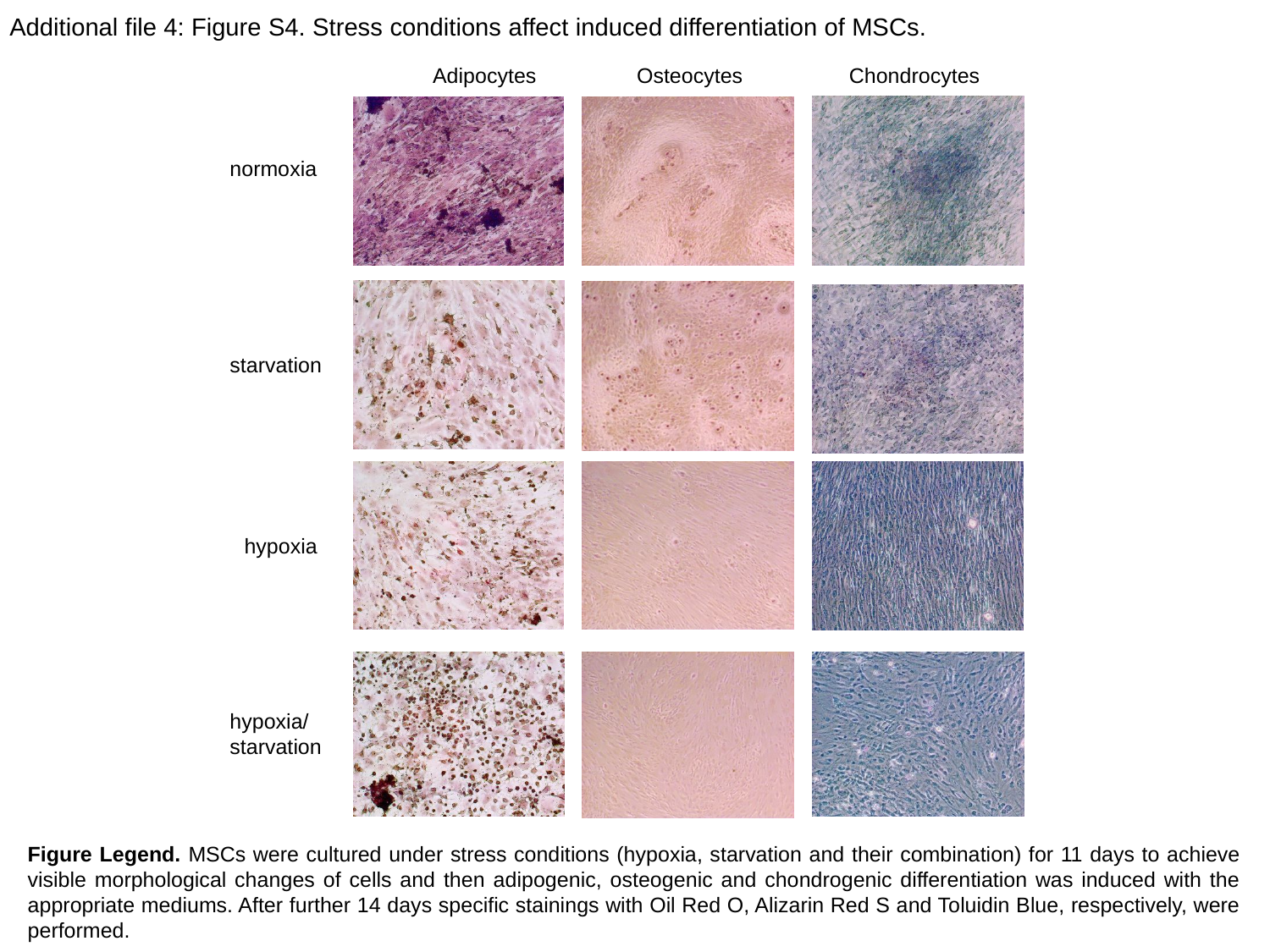

# Additional file 4: Figure S4. Stress conditions affect induced differentiation of MSCs.
 Adipocytes Osteocytes Chondrocytes
normoxia
starvation
hypoxia
hypoxia/
starvation
Figure Legend. MSCs were cultured under stress conditions (hypoxia, starvation and their combination) for 11 days to achieve visible morphological changes of cells and then adipogenic, osteogenic and chondrogenic differentiation was induced with the appropriate mediums. After further 14 days specific stainings with Oil Red O, Alizarin Red S and Toluidin Blue, respectively, were performed.
