# Supplementary material for: Chronic stress induces CD99, suppresses autophagy, and affects spontaneous adipogenesis in human bone marrow stromal cells
Source: Stem Cell Res Ther. 2017 Apr 18;8:83. doi: 10.1186/s13287-017-0532-3 (PMC5395812; doi:10.1186/s13287-017-0532-3)
Supplement: Supplementary file 5 — Starvation blocks induced adipogenesis of primary stromal cells. Prolonged stress (starvation) blocks induced adipocyte differentiation of primary stromal cells. ORO staining (lower panel) was performed at day 21. Representative pictures of four independent experiments. (PPTX 3141 kb) [file 13287_2017_532_MOESM5_ESM.pptx]

## Slide 1
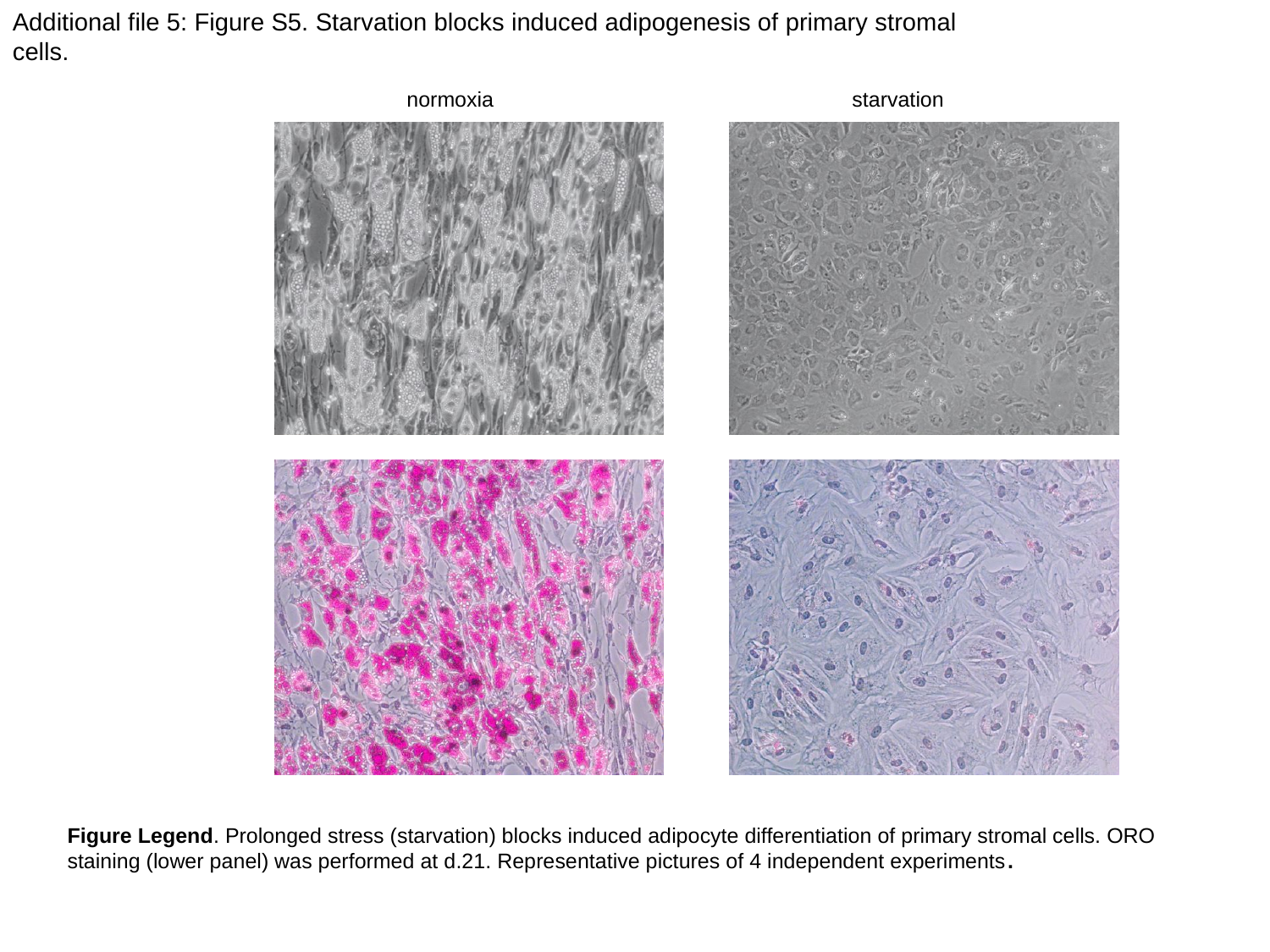

Additional file 5: Figure S5. Starvation blocks induced adipogenesis of primary stromal cells.
starvation
normoxia
Figure Legend. Prolonged stress (starvation) blocks induced adipocyte differentiation of primary stromal cells. ORO staining (lower panel) was performed at d.21. Representative pictures of 4 independent experiments.
