# Supplementary material for: Chronic stress induces CD99, suppresses autophagy, and affects spontaneous adipogenesis in human bone marrow stromal cells
Source: Stem Cell Res Ther. 2017 Apr 18;8:83. doi: 10.1186/s13287-017-0532-3 (PMC5395812; doi:10.1186/s13287-017-0532-3)
Supplement: Supplementary file 6 — Bafilomycin A1 treatment of MSCs. (A) hTERT-positive MSCs were starved for 3 days and treated with bafilomycin A1 for the last 3 hours. Pictures are representative of three independent experiments. (B) MSCs treated with bafilomycin A1 were subjected to WB to detect autophagy-involved proteins. (PPTX 1761 kb) [file 13287_2017_532_MOESM6_ESM.pptx]

## Slide 1
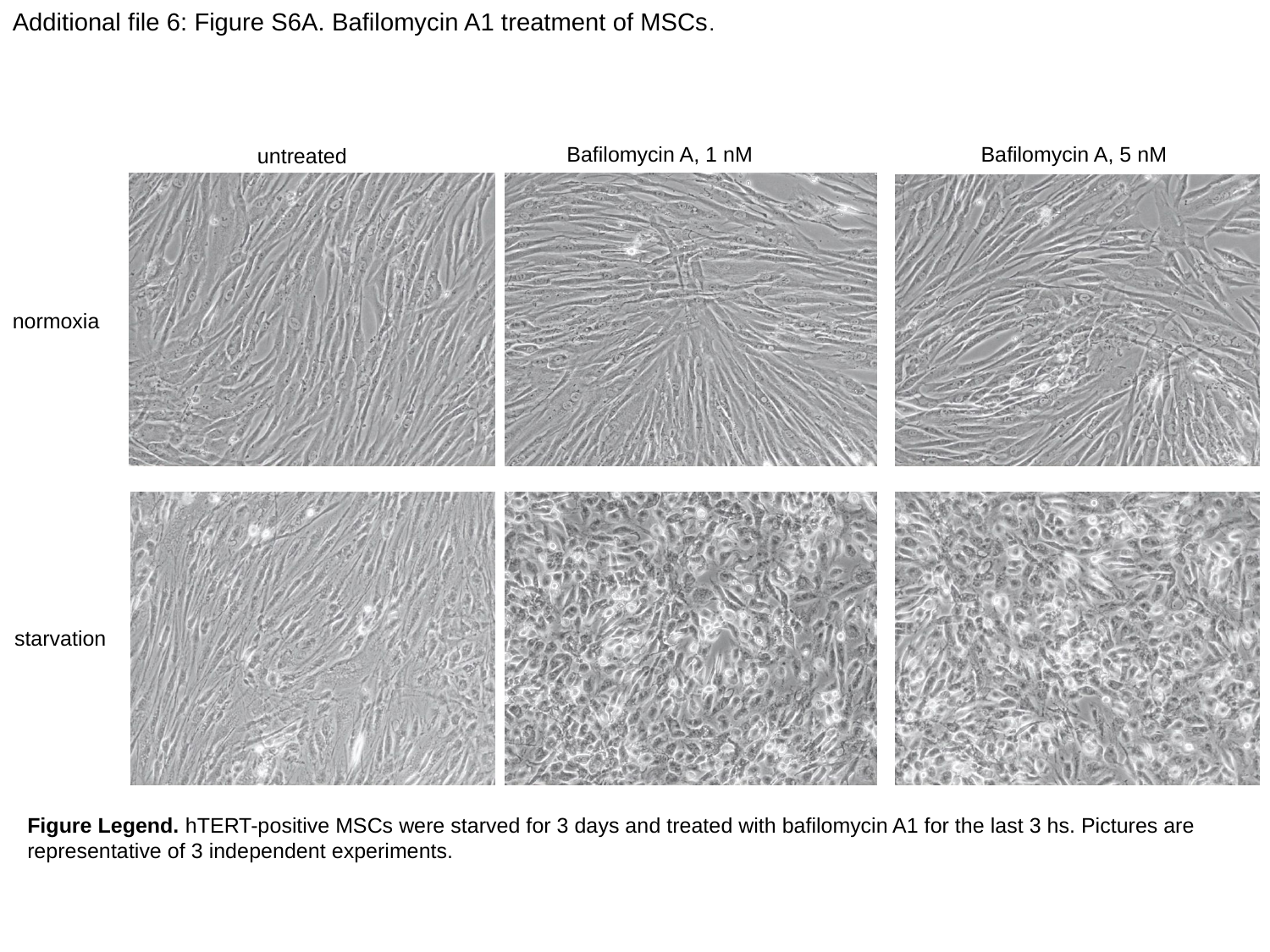

Additional file 6: Figure S6A. Bafilomycin A1 treatment of MSCs.
Bafilomycin A, 5 nM
Bafilomycin A, 1 nM
untreated
normoxia
starvation
Figure Legend. hTERT-positive MSCs were starved for 3 days and treated with bafilomycin A1 for the last 3 hs. Pictures are representative of 3 independent experiments.

## Slide 2
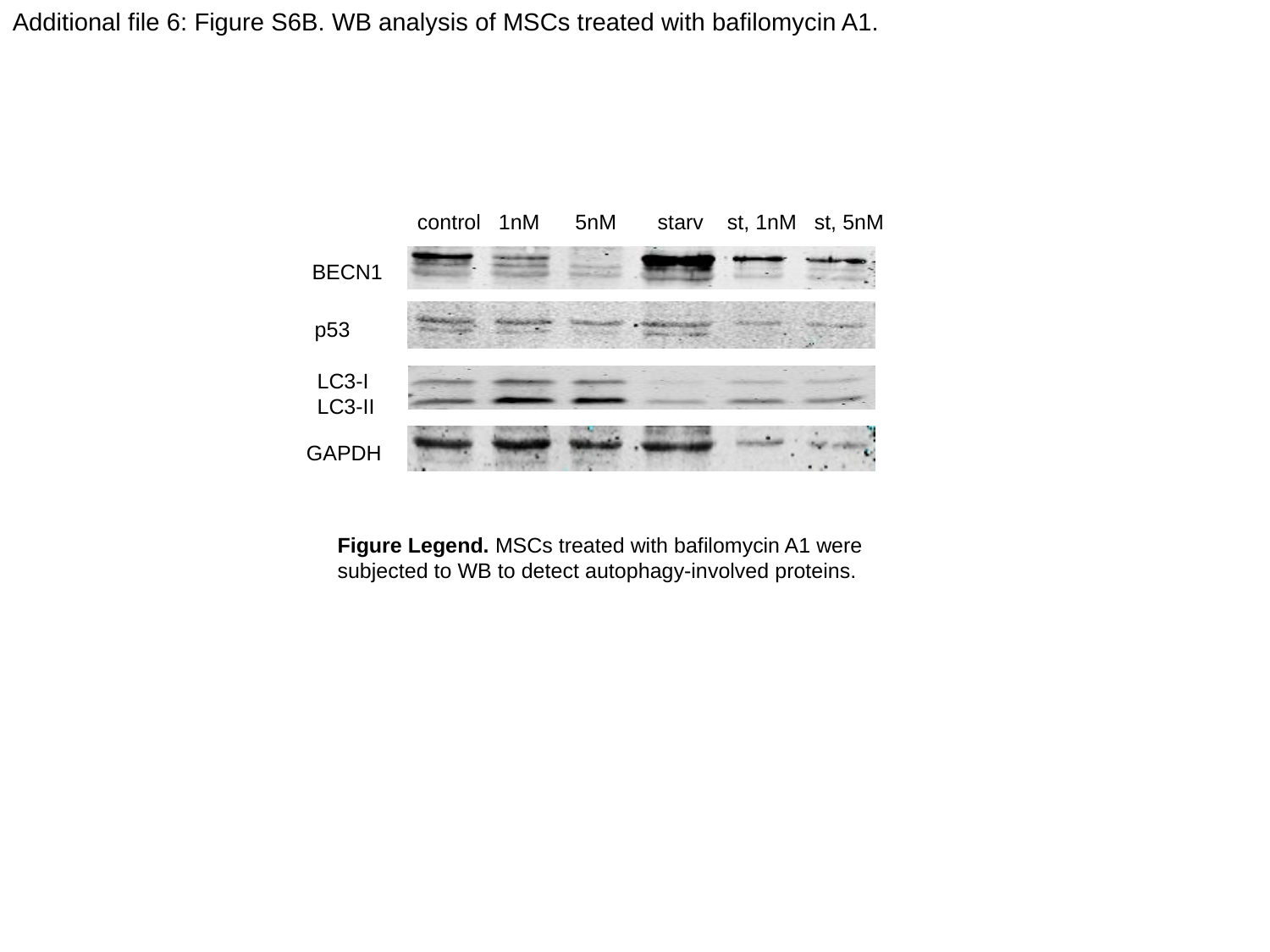

Additional file 6: Figure S6B. WB analysis of MSCs treated with bafilomycin A1.
 control 1nM 5nM starv st, 1nM st, 5nM
BECN1
p53
LC3-I
LC3-II
GAPDH
Figure Legend. MSCs treated with bafilomycin A1 were subjected to WB to detect autophagy-involved proteins.
